# Gaming behavior and brain activation using functional near‐infrared spectroscopy, Iowa gambling task, and machine learning techniques

**DOI:** 10.1002/brb3.2536

**Published:** 2022-03-15

**Authors:** Denis Kornev, Stanley Nwoji, Roozbeh Sadeghian, Saeed Esmaili Sardari, Hadis Dashtestani, Qinghua He, Amir Gandjbakhche, Siamak Aram

**Affiliations:** ^1^ Information System Engineering and Management Program Harrisburg University of Science and Technology Harrisburg Pennsylvania USA; ^2^ Data Analytics Program Harrisburg University of Science and Technology Harrisburg Pennsylvania USA; ^3^ Computer and Information Systems Program Harrisburg University of Science and Technology Harrisburg Pennsylvania USA; ^4^ Eunice Kennedy Shriver National Institute of Child Health and Human Development National Institutes of Health Bethesda Maryland USA; ^5^ Department of Psychology Southwest University Chongqing China

**Keywords:** cognitive neuroimaging, functional near‐infrared spectroscopy, Iowa gambling task, machine learning

## Abstract

**Introduction:**

The current study investigates the utilization and performance of machine learning (ML) algorithms in the cognitive task of finding the correlation between numerical parameters of the human brain activation during gaming. We hypothesize that our integrated feature extraction platform is able to distinguish between different psychosomatic conditions in the gaming process as measured by the functional near‐infrared brain imaging technique.

**Methods:**

For demonstration, the decision‐making process was constructed in the experiment environment that combined gaming simulator, such as the Iowa Gaming Task (IGT), with functional near‐infrared spectroscopy (fNIRS) as the neuroimaging technique. Features of fNIRS levels were extracted, averaged, and synchronized by time with the IGT dataset to predict the task score inside ML algorithms, such as multiple regression, classification and regression trees, support vector machine, artificial neural network, and random forest. For findings validation, the experiment data were resampled by training and testing sets. Further, a training dataset was used to train the ML algorithms, and prediction accuracy was estimated by repeated cross‐validation methods and compared by R squared and root mean square error (RMSE). The model with the best accuracy was used with the testing dataset and finalized the experiment.

**Results:**

During the experiment, the highest correlation was identified in the fourth block between the oxy‐hemoglobin signal and IGT score in average value (0.24) and signal feature (0.57). Such relationship is due to block 4 characterization as “conceptual” period when participants task experience reaches the maximum, and rewards raise accordingly. Simultaneously, ML algorithms, constructed based on training data set, demonstrate acceptable performance, and RMSE as the primary performance metric dynamically increases from block 1 to block 5, from the state of uncertainty and unknown to the certainty and risky. In contrast, R squared decreases during the same transition. In most IGT blocks, the best fitted model was determined as support vector machine with radial bases function kernel, and predictions were made with the highest accuracy (lowest RMSE) than in training models.

**Conclusion:**

Obtained findings showed the applicability and capability of ML models as a powerful technique to evaluate the cognitive neuroimaging task result. Moreover, in terms of features it was identified that the hemodynamic response reacts to the acceleration decision‐making process and raises more significance than it was observed before.

## Graphical abstract

The current study investigates the utilization and performance of machine learning (ML) algorithms in the cognitive task of finding the correlation between parameters of the human brain activation during gaming. We hypothesize that our integrated feature extraction platform is able to distinguish between different psychosomatic conditions in the gaming process as measured by the functional near‐infrared brain imaging technique.

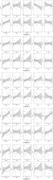

## 1 INTRODUCTION

Gaming addiction has become one of the most critical dimensions of modern society. It is not surprising as gamer auditory demonstrates a positive trend year by year. Accordingly, Entertainment Software Association reports that about 65% of the US adult population regularly plays video games (ESA, 2019). In other words, 164 million Americans build their daily decision‐making activity in accordance with a changeable game environment (Palaus et al., 2017). In this case, clinicians, researchers, and practitioners are forced to create new methods and instruments to classify new diseases and identify characteristics and treatments. Moreover, the American Medical Association (AMA) and the World Health Organization (WHO) added “game disorder” in their official manuals and classifications (APA, 2018; WHO, 2018). Thus, they were established characteristics of “new phenomenon” (Przybylski et al., 2017), inclusion limits, and symptoms such as anxiety, anger, and depression when the game is taken away, having problems at work, at school, and in family life, loss of interest in everyday activity and habits, and losing the power to resist gaming (APA, 2018). Losing behavioral control, people addicted to the game perceive the game as a necessity, meeting the need for a short‐term instant risk without long‐term planning. To distinguish patients by their risk‐acceptance strategy and sort them by the category of addicted and healthy, Bechara and Damasio (2002) adopted an earlier developed gaming simulator, called Iowa Gambling Task (IGT).

IGT was originally developed in 1994 to classify patients with damages of the ventromedial prefrontal cortex (Bechara et al., 1994). At the beginning of the task, the experiment participant receives $2000 virtual money and can increase or decrease this amount during the subsequent 100 card selection trials from four decks. Two decks (A and B) have a high probability of gaining or losing and are not preferred in the scope perspective. They are “bad” decks. The other two decks (C and D) have a low probability of gaining or losing and are preferred from the perspective. They are “good” decks. The score of IGT calculates the difference between the sum of a selection of advantageous (“good”) decks and disadvantageous (“bad”) decks. It is assumed that healthy participants’ experience will increase while performing the task, and the number of choosing cards from the “good” decks will be higher than the number of selecting cards from the “bad” decks. Thus, the IGT score will increase accordingly (Bechara & Damasio, 2004).

Another dimension of IGT is five blocks that conditionally divided 100 trials by 20 trails in each block. These blocks have a different psychiatric meaning. During the first 20 trials (block 1), experiment participants are in the condition of total unknown and uncertainty about the degree of probability rewards or punishment assigned by the card decks and make a decision without any strategy, resulting in money‐losing. This is the “pre‐punishment” period (Bechara & Damasio, 2004). Somewhere during the second 20 trials (block 2), participants begin to understand the general distribution of advantageous decisions between decks, and their understanding and knowledge continue to increase trial by trial through the third 20 choices. These periods are called “pre‐hunch” and “hunch” (Bechara & Damasio, 2004). By the fourth 20 trials (block 4), participants prove their hypothesis about gain probability and develop different tactics and strategies to win as much as possible and test it during the last 20 trials (block 5). These two final conditions are the “conceptual” period and “risky” state (Bechara & Damasio, 2004).

Despite more than 20 years of history, IGT does not lose its relevance even now. On the contrary, combined with functional neuroimaging techniques, such as electroencephalography (EEG), functional magnetic resonance imaging (fMRI), or functional near‐infrared spectroscopy (fNIRS), it becomes a powerful instrument for human brain cognitive exploration and analysis (Aram et al., 2019)

Historically, the task of investigating gaming patterns and their effect on human brain activity has been solved by collecting data from neuroimaging devices, such as PET and EEG in the form of an electrical signal and their analysis using statistical methods: general linear model, analysis of variance (ANOVA), *t*‐test, and correlation coefficient (Friston et al., 1995). By 2017, about 60% of neuroimaging‐related experiments were performed with fMRI techniques (Palaus et al., 2017). The fNIRS method based on the Beer–Lambert equation was introduced in 1988 (Delpy et al., 1988). This relatively new neuroimaging technique has several substantial straights, such as the ability to provide measuring in motions, portability, and inexpensively (Scarapiccha et al., 2017). Following fNIRS physics, optical light transmission, absorption, and scattering through human head tissue allow for registered changes of oxy‐hemoglobin (HbO) and deoxy‐hemoglobin (HbR) levels in target regions of interest (ROI) (Quaresima & Ferrari, 2019).

Despite the achieved success in dealing with functional imaging data analysis, statistical parametric mapping, and examining the brain regions in dynamics and different conditions, several limitations constrain statistical methods’ applicability. So, statistical methods are driven by mathematical and probabilistic long‐standing inferences and failing at a low degree of freedom. In this case, more powerful machine learning (ML) techniques, driven by data, are suitable for self‐learning based on the integrated behavioral model without outside interventions (Davatzikos, 2019) and are becoming more common in different cognitive neuroimaging tasks, such as pattern recognition, synchronization, and classification, experiment result evaluation, diagnoses prediction, spatiotemporal filtering, and so on (De Bruijne, 2016; Lemm et al., 2011). Moreover, ML is based on “wide data” and more abundant in predictive pattern generalization and suitable in the less controlled experiment environment, requiring minimal data adjustments and assumptions (Bzdok et al., 2018). If a correlation as a statistical tool determines the power of interrelationship and impacts one variable on another, then ML algorithms are capable of building models of variables mutual behavior, constructing prognoses and predictions of this behavior, and fit to evaluate the result of correlation (Kumar & Chong, 2018). Moreover, constructed around variables feature space increases prediction power and indicates the fields in which the parameter's correlation reaches maximum values.

The current study examines ML approaches in the cognitive task of finding and evaluating the correlation between brain activation parameters during gaming. On the first cup of the scale was placed accelerated decision‐making score measured as a result of performing the IGT by the participants, and on the second cup of the scale was set correspondent and synchronized neuroimaging response as changes of HbO levels in the human prefrontal cortex (PFC), measured by fNIRS.

Scientists used IGT and fNIRS capabilities to statistically estimate human brain performance during decision‐making in different stages and conditions in various studies. Thus, using *t*‐test it was determined that differences in the brain activation of experiment participants with low IGT score are less statistically significant than participants with high IGT score in both brain hemispheres (Suhr & Hammers, 2010). Using ANOVA, the significance of differences in HbO changes between IGT blocks was identified in studies of Ono et al. (2015), Balconi et al. (2018), and Li et al. (2019). Bembich et al. (2014) and Kora Venu et al. (2021) identified that HbO dynamics changes are more significant during the first half of IGT than in the second half of the task. A weak negative correlation (−0.39) in the left PFC and (−0.38) in the right PFC was identified between depression levels and HbO signal during IGT in patients with bipolar disorder by Ono et al. (2015). Li et al. (2019) identified the difference in the left and right PFC activation during IGT but calculated the correlation between HbO and task score only inside five IGT blocks: weak positive in the blocks 2, 3, and 4 (0.36, 0.41, and 0.45 respectively), negligible negative in block 1 (−0.01), and negligible positive in the block 5 (0.09). The summary of provided works is presented in Table 1.

**TABLE 1:** Iowa gaming task (IGT) and fNIRS in neuroimaging. Literature review summary

Author	Experiment	Analytical method	Result
Suhr and Hammers (2010)	IGT, fNIRS, HbO	*t*‐test	Changes of HbO in the left PFC of participants with lower IGT score (*M* = −0.4, SD = 1.9) have significantly lower values than participants with hight IGT score (*M* = 0.3, SD = 1.7): *t*(53) = −1.27, *p*‐value = 0.21
			Changes of HbO in the right PFC of participants with lower IGT score (*M* = 0.3, SD = 1.2) have significantly lower values than participants with high IGT score (*M* = 1.4, SD = 1.8): *t*(53) = −1.98, *p*‐value = 0.05
Bembich et al. (2014)	IGT, fNIRS, HbO	*t*‐test	Changes of HbO are more significant during the first half of IGT than the second half
			There are significant differences in HbO levels between high and low‐risk IGT card choices
Ono et al. (2015)	IGT, fNIRS, HbO	ANOVA, Pearson correlation coefficient	Right PFC: *r* = −0.39 (p‐value = 0.040)
			Left PFC: *r* = −0.384 (p‐value = 0.044)
			The effect between IGT blocks is significant: *F*(5, 1) = 7.201, *p*‐value = 0.013
Balconi et al. (2018)	IGT, fNIRS, HbO	ANOVA	The effect between IGT blocks is significant: *F*(2, 50) = 8.54, *p*‐value ≤ 0.001, *η*2 = 0.41
Li et al. (2019)	IGT, fNIRS, HbO	ANOVA, Pearson correlation coefficient	Block 1: *r* = −0.009 (p‐value = 0.483)
			Block 2: *r* = 0.364 (p‐value = 0.037)
			Block 3: *r *= 0.409 (p‐value = 0.021)
			Block 4: *r* = 0.456 (p‐value = 0.011)
			Block 5: *r* = 0.090 (p‐value = 0.335)
			The effect between IGT blocks and brain hemispheres is significant:*F*(1, 24) = 6.27, *p*‐value = 0.02, *η*2 = 0.207
Kora Venu et al. (2021)	IGT, fNIRS, HbO	ANOVA	Changes of HbO in the left PFC have higher values than in the right PFC: F(1, 27) = 12.3, *p*‐value < 0.001, *η*2 = 0.056
			IGT score in Blocks 4 and 5 significantly higher than in Blocks 1, 2, and 3: *F*(3.03, 109.13) = 3.28, *p*‐value < 0.05, *η*2 = 0.05

Nevertheless, the possibility to use the ML mechanics in neuroimaging was successfully demonstrated and reviewed in various studies (Aram et al., 2020, 2021). But, despite the popularity and widespread use of ML algorithms in dealing with neuroimaging data, their application to combined IGT and fNIRS datasets is still poorly analyzed (Table 1). Moreover, methods of statistical analysis transfer gaps and uncertainties of their application to tasks of investigation dependences of the brain activity and gaming behavior: using a simple *t*‐test or ANOVA is less robust in the task of variables performance prediction, and found correlation cannot be evaluated in a dynamic environment (Bzdok et al., 2018; Kumar & Chong, 2018). Simultaneously, constructed inside the cognitive space, self‐learning ML models can predict decision making capabilities based on the HbO levels changes in the brain, and are suitable for evaluating their correlation with gaming behavior. Thus, the following “null” hypothesis could be set: There is no correlation between brain activation and gaming behavior. Alternative hypothesis is that there is a correlation between brain activation and gaming behavior, and this correlation has significantly different values in PFC hemispheres depending on the game stage, condition, and participant's psychosomatic condition.

The current paper aims to overcome the previously achieved results (Table 1) by spreading the correlation by features and makes a conclusion about the best ML model to predict gaming behavior by brain activation. By “the best” we mean the best ML algorithm, and the possibility to assess the ML approach's applicability in the particular cognitive task, as presented in the current work (Davatzikos, 2019).

## 2 METHODS

### 2.1 Participants and data

Data collection from 30 young adult volunteers (*N* = 30)—25 females and five males in the age range between 19 and 26 (*M* = 21.8, SD = 1.77)—was approved by the Southwest University Institutional Review Board (Chongqing, China). All participants signed informed consent (Li et al., 2019). All experiment participants were right‐handed, and they did not report any neurological or psychiatric health issues and problems with vision.

Demographic characteristics of the study population are presented in Table 2.

**TABLE 2:** Demographic characteristics of the study population

Experiment participants (*N *= 30)		
Parameter		Value
Female		25
Male		5
Age range	Years	19–26
Age mean	M	21.8
Age standard deviation	SD	1.77

All testees were randomly selected for the experiment without any coercion or violence. The chance to be chosen was equal for all of them. Participants were marked numerically from 002 to 031 without any forms of individuality.

As mentioned before, the target variable is the IGT score, measured numerically, predicted by levels of HbO changes, and divided by five blocks following classic IGT rules (Bechara et al., 1994). The same IGT score will be analyzed separately for the left and right brain hemispheres in each block.

**FIGURE 1:**
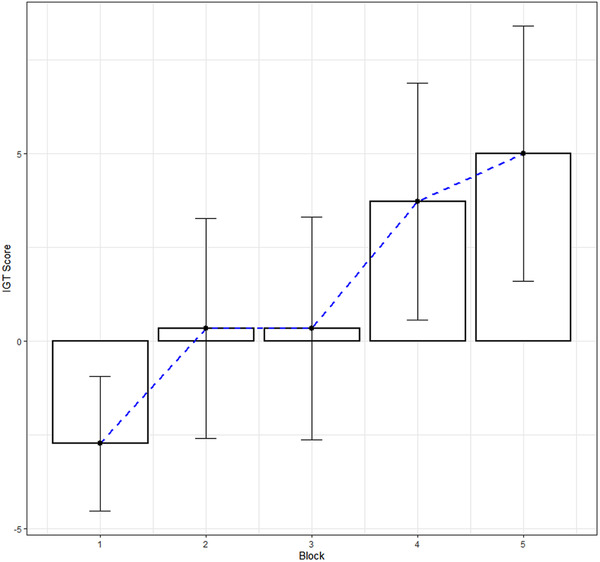
IGT score distribution with mean value and error bars

Central tendencies of the research IGT score are presented in Table 3. Each of the five blocks is a set of 30 numbers that are individual scores of participants achieved in each block. Key data parameters demonstrate positive dynamics, increase during the task: sum from −82.00 in block 1 to 150.00 in block 5; mean from −2.73 in block 1 to 5.00 in block 5; and median from −2.00 in block 1 to 5.00 in block 5. Such tendency indicates that participants’ experience increases from the beginning to the end of the task, which is typical for healthy people who are experiment testees. The distribution of the IGT score by five blocks is presented in Figure 1. Meanwhile, the standard error of a sample mean (SE.mean) increases from 0.87 in block 1 to 1.66 in block 5; the confidential interval that consist of 95% of mean true values (CI.mean.0.95) increases from 1.79 in block 1 to 3.40 in block 5; variance increases from 23.02 in block 1 to 83.24 in block 5; and standard deviation increases from 4.79 in block 1 to 9.12 in the block 5 that indicates activation of risk factor: participants use different strategies and tactics to gain as much as possible, and with the increase of experience the risk acceptability raises proportionally.

**TABLE 3:** Dependent variable descriptive analysis

	Block 1	Block 2	Block 3	Block 4	Block 5
Parameter	IGT score	IGT score	IGT score	IGT score	IGT score
nbr.val	30.0000	30.0000	30.0000	30.0000	30.0000
nbr.null	7.00000	9.00000	4.00000	6.00000	0.00000
nbr.na	0.00000	0.00000	0.00000	0.00000	0.00000
min	−20.0000	−20.0000	−18.0000	−18.0000	−16.0000
max	6.00000	20.0000	20.0000	20.0000	20.0000
range	26.0000	40.0000	38.0000	38.0000	36.0000
sum	−82.0000	10.0000	10.0000	112.000	150.000
median	−2.00000	0.00000	−1.00000	2.00000	5.00000
mean	−2.73333	0.33333	0.33333	3.73333	5.00000
SE.mean	0.87616	1.43546	1.45455	1.54543	1.66575
CI.mean.0.95	1.79196	2.93584	2.97488	3.16076	3.40684
var	23.0299	61.8161	63.4713	71.6506	83.2414
std.dev	4.79895	7.86232	7.96689	8.46467	9.12367

On the opposite side of the correlation scale is HbO data collected by fNIRS and grouped by channels and hemispheres. This brain hemodynamics is the independent variable, reflecting participant's decisions as changes of levels—friction around zero between −1.5 and 5.5. In the current study, fNIRS data come preprocessed, that is, filtered and cleaned from artifacts.

### 2.2 Experimental design

**FIGURE 2:**
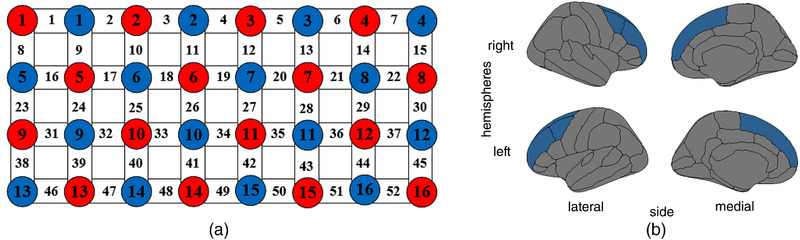
Experiment functional near‐infrared spectroscopy (fNIRS) channels arrangement on the regions of interest (ROI): (a) the probe/channel scheme (red—transmitters, blue—receivers, white—channels), (b) target ROI on the left and the right brain hemispheres

During the experiment, the computer version of the “classic” IGT experiment paradigm with 100 trials divided by five blocks was used (Bechara & Damasio, 2004; Bechara et al., 1994). Multichannel functional near‐infrared spectrometer FOIRE‐3000/16 (Shimadzu Corp., Japan) was used to collect fNIRS data that were further preprocessed and cleaned from artifacts and noise by wavelet‐minimum description length (Wavelet‐MDL) detrending algorithm from NIRS‐SPM software package (Ye et al., 2009). On the head of participants was placed a special cap with 32 integrated probs (16 transmitters and 16 receivers), which covered the PFC, and was connected by 52 channels that propagate light beam with continuous wavelengths of 780, 805, and 830 nm, and allowed recording of signals with a 4 Hz frequency. The probs and channels’ position was standardized for each participant using Montreal Neurological Institute (MNI) space and International Positioning System (Li et al., 2019). A three‐dimensional (3D) digitizer was used to record the positioning of optical channels and probs on the participant's head with five landmarks: nasion point (Nz), inion point (Iz), central zero (Cz), left (AL), and right (AR) preauricular points. For the result reliability and stability, target ROI of the brain were covered by channels 28, 35, 36, 42, and 43 for the left PFC and channels 25, 32, 33, 40, and 41 for the right PFC (Figure 2; Table 4).

**TABLE 4:** Cortical channels localization, mm withing ROI in MNI space by the left (LH) and right (RH) brain hemispheres

Anatomical label	Channel	MNI Coordinates			
		*x*	*y*	*z*	SD
LH	28	−23	62	27	7.0
	35	−16	71	17	5.4
	36	−36	60	17	6.0
	42	−9	73	9	7.7
	43	−27	68	9	5.7
RH	25	33	59	25	7.3
	32	43	59	14	5.4
	33	26	69	16	5.7
	40	39	64	5	5.1
	41	20	73	7	5.0

**FIGURE 3:**
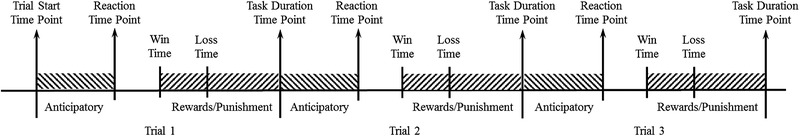
IGT experiment design with fNIRS synchronization time points

The synchronization of the IGT experiment and HbO signal recording from the fNIRS was started after 20 s from the beginning of the test that was spent on the equipment warm‐up at the trial start time point (Figure 3). During the following anticipatory interval, the experiment participant made their decision about card choice. The hemodynamic response is recorded at the Reaction Time Point (RTP); levels of HbO from five channels on the left‐brain hemisphere and five channels from the right hemispheres are averaged and logged. There is a short period of time (about 0.55 s) after card selection and before receiving information about gaining virtual money. If money was lost, then the information about the appropriate amount appeared about 1.5 s after details about the winning amount. Thereby, the time interval between information about the winning amount and the trial's end is the rewards/punishment period. The hemodynamic response is recorded at the task duration time point (DTP); levels of HbO from five channels on the left‐brain hemisphere and five channels from the right hemispheres are averaged and logged. Each IGT session's length was about 9 m, and the last 20 s of each session was spent on information accounting.

RTP and DTP are boundaries of the anticipatory time interval and determine the start and the end of the participant's card choosing decision‐making period. They are the experiment's target points. Corresponding to RTP and DTP fNIRS signal measurements will be extruded from the whole dataset for the subsequent preprocessing, features extraction, and evaluation by ML models.

### 2.3 Features extraction

For improving the ML algorithm's performance and application, the set of features was built around the fNIRS signal. To avoid losing temporal information in the frequency domain, the set of features was extracted from averaged time courses of HbO records of the left and right brain hemispheres collected at two time points—RTP and DTP from IGT decision making time window: (a) mean (LHmean and RHmean) – HbO signal mean value; (b) variance (LHvar and RHvar) – the average error between mean and real HbO level; (c) standard deviation (LHsd and RHsd) – square root from variance; (d) kurtosis (LHku and RHku) – the shape of the HbO signal distribution in the vertical axes; (e) skewness (LHsk and RHsk) – the shape of the HbO signal distribution in the horizontal axis.

The correlation coefficients between HbO signal features and IGT score, measured by two methods—Pearson's product‐moment (Pearson, 1895) and Spearman's rank (Spearman, 1904) for the left and the right brain hemispheres and in each block presented in Table 5 and Figure 4. If Pearson's coefficient is best suited in normally distributed datasets, then Spearman's test is more robust and shows the best performance with skewed data (Mukaka, 2012; Schober et al., 2018).

**TABLE 5:** Correlation coefficients between HbO signal features and IGT score by the left (LH) and right (RH) brain hemispheres in five IGT blocks. (a) Pearson's product‐moment correlation; (b) Spearman's rank correlation

	Block 1		Block 2		Block 3		Block 4		Block 5	
Features	LH	RH	LH	RH	LH	RH	LH	RH	LH	RH
mean	0.36	0.34	0.30	0.28	0.25	0.25	0.48	0.44	0.30	0.33
std.dev	0.09	0.09	0.24	0.23	0.25	0.34	0.20	−0.04	0.00	0.02
variance	0.01	0.04	0.24	0.24	0.24	0.33	0.22	−0.02	0.04	0.06
kurtosis	−0.45	0.04	−0.18	−0.16	0.28	0.06	−0.16	0.21	0.01	0.14
skewness	−0.22	−0.12	0.03	−0.02	−0.13	−0.18	0.48	0.30	0.21	−0.13
*Average*	−0.04	0.07	0.12	0.11	0.17	0.16	0.24	0.17	0.11	0.08
A										

	Block 1		Block 2		Block 3		Block 4		Block 5	
Features	LH	RH	LH	RH	LH	RH	LH	RH	LH	RH
mean	0.48	0.45	0.22	0.20	0.24	0.25	0.39	0.39	0.25	0.33
std.dev	−0.02	0.10	0.27	0.23	0.19	0.28	0.09	−0.01	−0.08	−0.12
variance	−0.02	0.10	0.27	0.23	0.19	0.28	0.09	−0.01	−0.08	−0.12
kurtosis	−0.42	0.04	−0.21	−0.22	0.13	0.14	−0.09	0.20	0.04	0.19
skewness	−0.33	−0.09	−0.05	0.07	−0.17	−0.38	0.57	0.30	0.24	−0.15
*Average*	−0.06	0.12	0.10	0.10	0.11	0.11	0.21	0.17	0.07	0.02
B										

FIGURE 4The correlation between oxy‐hemoglobin (HbO) signal features and IGT score by blocks and the brain hemispheres: (a) Block 1, (b) Block 2, (c) Block 3, (d) Block 4, and (e) Block 5

**Figure:**
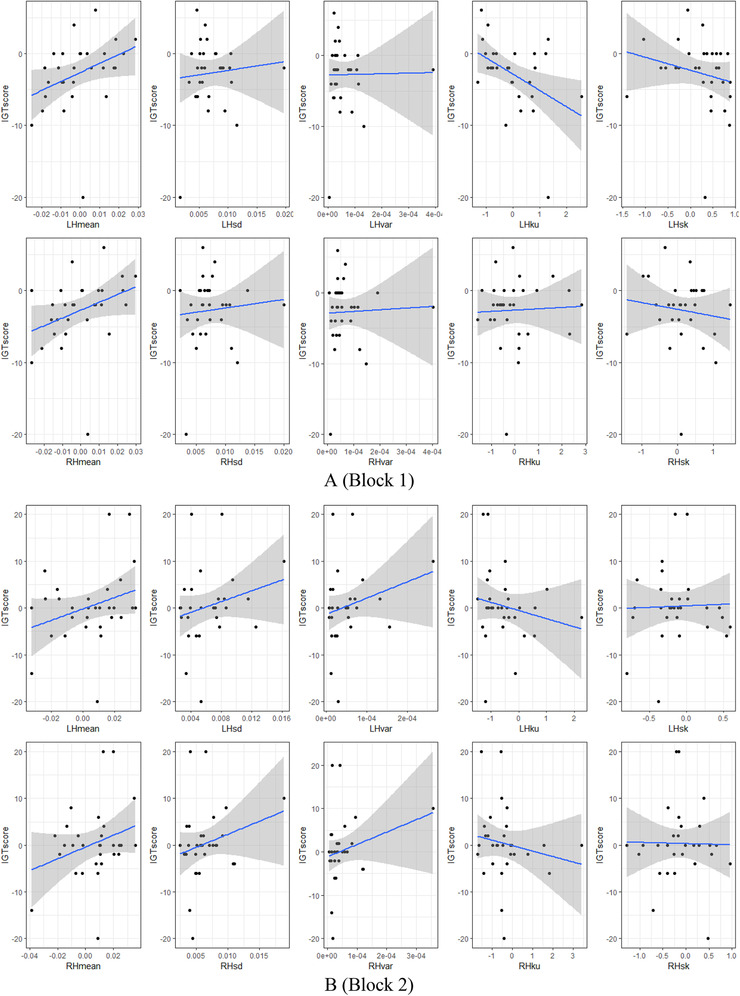

**Figure:**
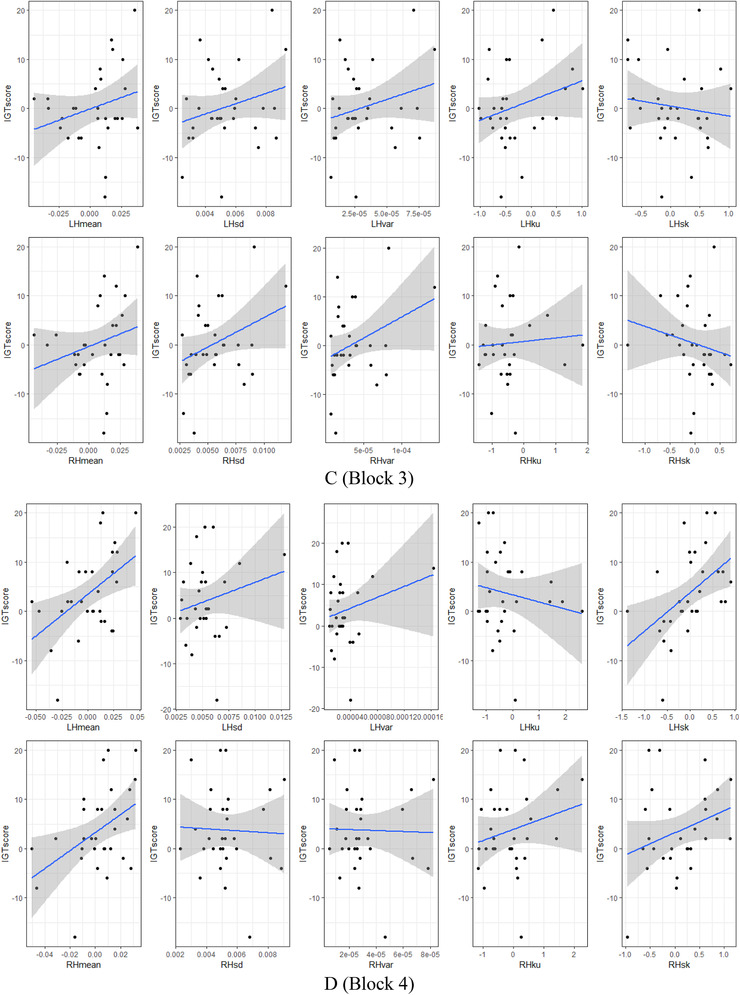

**Figure:**
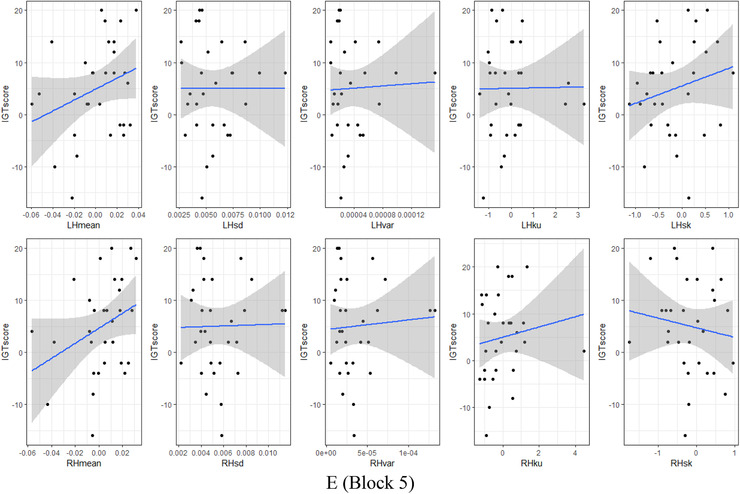

### 2.4 ML models

ML algorithms’ application to evaluate the correlation between experiment variables was performed in R 4.0.3 (R Core Team, 2018). Five techniques were run: multiple regression, classification and regression trees (CART), artificial neural network (ANN), support vector machine (SVM), and random forest. For achieving algorithms’ performance maximization, the task was repeated two times, and then the algorithm with the best accuracy was used to predict the IGT score.

First, the dataset of the HbO signal features from the left and the right brain hemispheres was normalized and split by training and testing in proportions of 70/30. Then training sample was used to train ML models, and the prediction accuracy was compared by two metrics: root mean squared error (RMSE) and coefficient of determination (R squared). Following Lemm et al. (2011), we described the most commonly used ML model validation techniques for brain imaging analysis, and validated the result by repeated fivefold cross‐validation (CV) method (Tables 5a and 6a).

Second, the training dataset was split in proportions of 80/20, and the result was validated by repeated 10‐fold CV. Moreover, other improvements were implemented as follows: switching the SVM kernel from linear to Gaussian radial bases function (RBF) and changing the number of trees in random forest from 100 to 500. Achieved RMSE and R squared are presented in Tables 6b and 7b.

**FIGURE 5:**
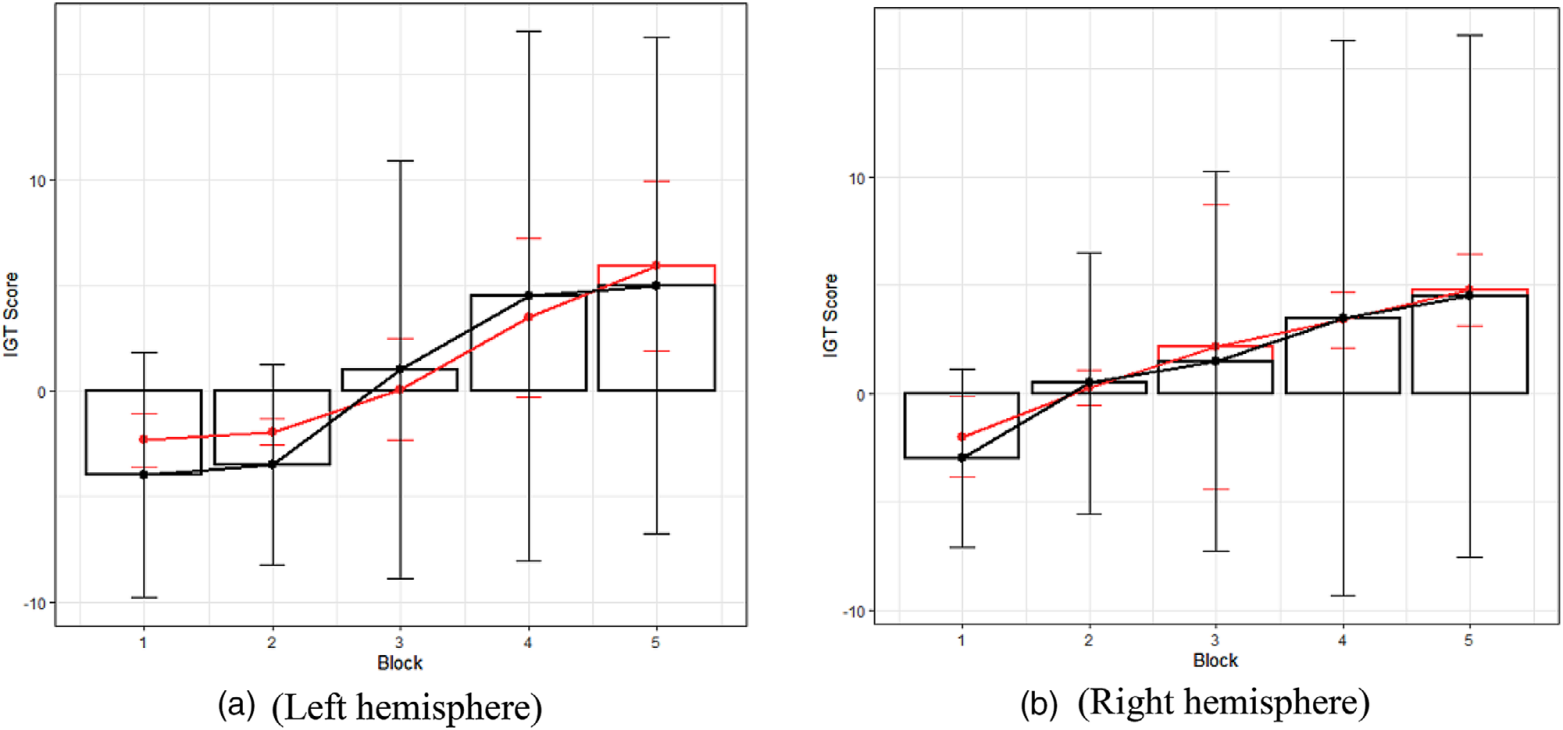
ML algorithms prediction accuracy; actual values (black) and predicted values (red): (a) the left hemisphere, (b) the right hemisphere

Lastly, the ML algorithm with the lowest RMSE measured in IGT units and R squared closest to 1 was applied to each block's testing dataset and in both hemispheres (Table 8). All five models were utilized separately. Thus, decision‐making behavior was predicted, measured by IGT score in different psychiatric states as a response to hemodynamic changes in the form of HbO signal. IGT score prediction distribution by five blocks and both brain hemispheres applied to the randomly selected testing data sample presented in Figure 5.

## 3 RESULT

### 3.1 Correlation

During the first 20 trials, the card selection decisions of experiment participants are erratic and random. This uncertainty is reflected in the negligible and statistically not significant correlation between brain activity and IGT score: the average Pearson's coefficient in block 1 is −0.04 (*t*(4) = −0.304, *p*‐value = 0.775) for the left hemisphere and 0.07 (*t*(4) = 1.047, *p*‐value = 0.354) (Table 5a). The average Spearman's rank as more robust demonstrates the strongest but still statistically not significant result: −0.06 (*t*(4) = −0.393, *p*‐value = 0.714) for the left hemisphere and 0.12 (*t*(4) = 1.340, *p*‐value = 0.251) for the right hemisphere (Table 5b). In terms of feature space, the Pearson's coefficient was spread between −0.45 and 0.36 for the left hemisphere and between −0.12 and 0.34 for the right hemisphere (Table 5a; Figure 4a). The Spearman's rank is as follows: from −0.42 to 0.48 for the left hemisphere and from −0.09 to 0.45 for the right hemisphere (Table 5b; Figure 4a). In block 1, the IGT score correlates more significantly with the HbO signal mean value of 0.48 for the left hemisphere and 0.45 for the right hemisphere (Table 5).

The participants have more knowledge in IGT block 2, therefore the HbO activity increases in their brains, and correlation grows to weakly positive accordingly but is still not statistically significant: the average Pearson's coefficient is 0.12 (*t*(4) = 1.412, *p*‐value = 0.2306) in the left hemisphere and 0.11 (*t*(4) = 1.316, *p*‐value = 0.258) in the right hemisphere (Table 5a). The average Spearman's rank generally repeats such numbers: 0.10 (*t*(4) = 1.023, *p*‐value = 0.363) in the left hemisphere and 0.10 (*t*(4) = 1.189, *p*‐value = 0.30) in the right hemisphere (Table 5, B). Spreading by features, the Pearson's coefficient reaches values between −0.18 and 0.30 for the left hemisphere and between −0.16 and 0.28 for the right hemisphere (Table 5a, Figure 4b); Spearman's rank generally repeats such numbers: from −0.21 to 0.27 for the left hemisphere and from −0.22 to 0.23 for the right hemisphere (Table 5b; Figure 4b). The highest achieved correlation values in block 2 reach 0.3 for the left hemisphere, and 0.28 for the right hemisphere by HbO signal mean value (Table 5).

In the middle of the task (block 3), the participant's experience continues to grow, and along with it the statistical significance of correlation also increases: the average Pearson's coefficient is 0.17 (*t*(4) = 2.303, *p*‐value = 0.082) in the left hemisphere and 0.16 (*t*(4) = 1.620, *p*‐value = 0.180) in the right hemisphere (Table 5a). The average Spearman's rank: 0.11 (*t*(4) = 1.576, *p*‐value = 0.190) in the left hemisphere and 0.11 (*t*(4) = 0.903, *p*‐value = 0.417) in the right hemisphere (Table 5b). By features, the Pearson's coefficient values are between −0.13 and 0.28 for the left hemisphere and between −0.18 and 0.34 for the right hemisphere (Table 5a; Figure 4c). Spearman's rank values range from −0.17 to 0.24 for the left hemisphere and from −0.38 to 0.28 for the right hemisphere (Table 5b; Figure 4c). The signal shape kurtosis performs the highest correlation, 0.28 for the left hemisphere and the signal skewness −0.38 for the left hemisphere (Table 5).

The “conceptual” period, that is, the threshold moment in the task, begins in the fourth set of 20 trials. Participants start to gain rewards, and hemodynamic in their brains correlates positively with increased IGT score: the average Pearson's coefficient is 0.24 (*t*(4) = 2.072, *p*‐value = 0.106) in the left hemisphere and 0.17 (*t*(4) = 1.923, *p*‐value = 0.126) in the right hemisphere (Table 5a). The average Spearman's rank value is 0.21 (*t*(4) = 1.772, *p*‐value = 0.151) in the left hemisphere and 0.17 (*t*(4) = 2.150, *p*‐value = 0.097) in the right hemisphere (Table 5, B). Dividing by features set, the Person's coefficient reaches values between −0.16 and 0.48 for the left hemisphere and between −0.04 and 0.44 for the left hemisphere (Table 5a; Figure 4d); Spearman's rank reaches values between −0.09 and 0.57 for the left hemisphere and between −0.01 to 0.39 for the right hemisphere (Table 5b; Figure 4d). The correlation between IGT score and HbO signal skewness is the highest achieved result for the left hemisphere 0.57 and mean value for the right hemisphere 0.44 (Table 5).

In the last set of 20 “risky” trials, participant's total money gain increases, but brains hemodynamic reaches positive and negative peak values that reflect a decelerating of correlation growth: the average Pearson's coefficient is 0.11 (*t*(4) = 1.852, *p*‐value = 0.137) in the left hemisphere and 0.08 (*t*(4) = 1.112, *p*‐value = 0.328) in the right hemisphere (Table 5, A). The average Spearman's rank: 0.07 (*t*(4) = 1.011, *p*‐value = 0.369) in the left hemisphere and 0.02 (*t*(4) = 0.264, *p*‐value = 0.804) in the right hemisphere (Table 5, B). In spreading by HbO signal features: the Person coefficient reaches values between 0 and 0.3 for the left hemisphere and between −0.13 and 0.33 for the right hemisphere (Table 5a; Figure 4e); the Spearman's rank reaches values between −0.08 and 0.25 for the left hemisphere and −0.15 and 0.33 for the right hemisphere (Table 5b; Figure 4e). The HbO signal mean values demonstrate the highest achieved correlation result of 0.3 for the left hemisphere and 0.33 for the right hemisphere (Table 5).

### 3.2 Machine Learning algorithms performance

In the current study, ML algorithms serve to evaluate the correlation between parameters of brain hemodynamics and the decision‐making score inside the cognitive environment. Table 6 illustrates the performance of ML techniques measured by RMSE and evaluated by 70/30 holdout method and fivefolds CV (Table 6, A), and 80/20 holdout and 10‐fold CV (Table 6b). The following tendencies are in the transition of ML performance through five blocks in the left and the right hemispheres:

1. RMSE increases from block 1 to block 5 with fivefold CV and 70/30 holdout (from 5.860 (*t*(4) = 7.587, *p*‐value = 0.001) to 8.956 (*t*(4) = 25.914, *p*‐value < 0.001) in the left hemisphere and from 5.170 (*t*(4) = 11.615, *p*‐value < 0.001) to 9.056 (*t*(4) = 52.288, *p*‐value < 0.001) in the right hemisphere) (Table 6, A);
2. RMSE increases from block 1 to block 5 with 10‐fold CV and 80/20 holdout (from 4.762 (*t*(4) = 4.989, *p*‐value = 0.007) to 8.676 (*t*(4) = 25.041, *p*‐value < 0.001) in the left hemisphere and from 4.554 (*t*(4) = 7.936, *p*‐value = 0.001) to 8.622 (*t*(4) = 24.386, *p*‐value < 0.001) in the right hemisphere) (Table 6b);
3. RMSE decreases in each block and both hemispheres by switching validation methods from 70/30 holdout and fivefold CV to 80/20 holdout and 10‐fold CV (Table 6a, b);
4. R squared decreases from block 1 to block 5 with 10‐fold CV and 70/30 holdout (from 0.42 (*t*(2) = 6.851, *p*‐value = 0.020) to 0.19 (*t*(2) = 2.939, *p*‐value = 0.098) in the left hemisphere and from 0.306 *(t*(2) = 7.977, *p*‐value = 0.015) to 0.18 (*t*(2) = 2.71, *p*‐value = 0.113) in the right hemisphere) (Table 7a);
5. R Squared decreases from block 1 to block 5 with 10‐fold CV and 80/20 holdout (from 0.87 (*t*(2) = 39.955, *p*‐value < 0.001) to 0.82 (*t*(3) = 32.569, *p*‐value < 0.001) in the left hemisphere and from 0.89 (*t*(2) = 7.977, *p*‐value = 0.001) to 0.80 (*t*(3) = 67.634, *p*‐value < 0.001) in the right hemisphere) (Table 7b);
6. R Squared increases in each block and both hemispheres by switching validation methods from 70/30 holdout and fivefold CV to 80/20 holdout and 10‐fold CV (Table 7a, b);
7. In each IGT block, the best model is the model with the lowest RMSE (in units of IGT score) and highest R squared (SVM with RBF kernel in blocks 1, 3, and 5, Random Forest with 500 trees in block 4, ANN in block 2 in the left hemisphere, and SVM with RBF kernel in blocks 1, 4, and 5, ANN in block 2, Random Forest with 500 trees in the right hemisphere) (Table 8);
8. In the set of best fitted models, RMSE increases from block 1 to block 5 (Table 8);
9. All achieved best fitted ML algorithms are more accurate by achieved RMSE than training models in all IGT blocks and hemispheres (Tables 7; Table 8);
10. RMSE of best fitted models is lower than the standard deviation of the IGT score dataset (Table 3) in all blocks (Table 8).

**TABLE 6:** RMSE of ML algorithms by the left (LH) and right (RH) brain hemispheres in five IGT blocks: (a) 70/30 holdout, fivefolds CV; (b) 80/20 holdout, 10‐folds CV

	Block 1		Block 2		Block 3		Block 4		Block 5	
Algorithm	LH	RH	LH	RH	LH	RH	LH	RH	LH	RH
Multiple Regression	8.70	6.91	7.88	6.26	7.61	7.42	7.98	9.27	9.85	8.68
CART	4.85	4.71	7.18	5.31	6.88	7.01	7.72	7.95	8.08	8.91
ANN	5.71	4.92	6.89	4.09	7.07	7.05	7.83	8.61	8.60	9.71
SVM (*Linear Kernel)*	5.86	4.40	6.93	5.28	8.81	7.82	3.37	8.51	9.69	9.01
Random Forest *(100 Trees)*	4.18	4.91	7.27	5.40	7.00	6.11	7.13	7.97	8.56	8.97
*Average*	5.86	5.17	7.23	5.27	7.47	7.08	6.81	8.46	8.95	9.05
A										

	Block 1		Block 2		Block 3		Block 4		Block 5	
Algorithm	LH	RH	LH	RH	LH	RH	LH	RH	LH	RH
Multiple Regression	8.54	6.75	6.96	6.08	7.42	5.93	9.13	8.33	8.89	9.59
CART	3.81	4.33	6.25	4.52	6.61	5.22	7.17	7.75	8.44	7.92
ANN	4.24	4.17	5.04	4.06	5.96	5.60	7.69	7.76	8.89	8.90
SVM (*RBF Kernel)*	3.37	3.37	5.46	4.41	5.90	6.42	6.42	7.26	7.84	7.70
Random Forest *(500 Trees)*	3.85	4.15	5.90	5.08	7.01	4.78	6.24	7.77	8.32	9.00
*Average*	4.76	4.55	5.92	4.83	6.58	5.59	7.33	7.77	8.67	8.62
B										

**TABLE 7:** R Squared of ML algorithms by the left (LH) and right (RH) brain hemispheres in five IGT blocks: (a) 70/30 holdout, fivefolds CV; (b) 80/20 holdout, 10‐folds CV

	Block 1		Block 2		Block 3		Block 4		Block 5	
Algorithm	LH	RH	LH	RH	LH	RH	LH	RH	LH	RH
Multiple Regression	0.35	0.35	0.25	0.18	0.28	0.43	0.31	0.25	0.14	0.05
CART	NA	NA	0.25	0.13	NA	0.64	NA	NA	NA	NA
ANN	NA	NA	0.29	0.20	0.33	0.39	NA	NA	NA	NA
SVM (*Linear Kernel)*	0.55	0.23	0.39	0.41	0.17	0.27	0.35	0.24	0.12	0.24
Random Forest *(100 Trees)*	0.38	0.34	0.29	0.21	0.28	0.41	0.38	0.19	0.33	0.27
*Average*	0.42	0.30	0.29	0.22	0.26	0.42	0.34	0.22	0.19	0.18
A										

	Block 1		Block 2		Block 3		Block 4		Block 5	
Algorithm	LH	RH	LH	RH	LH	RH	LH	RH	LH	RH
Multiple Regression	0.83	0.86	0.84	0.82	0.79	0.44	0.79	0.83	0.84	0.82
CART	NA	NA	0.61	0.78	0.64	0.67	0.84	0.81	0.75	0.79
ANN	NA	NA	0.89	0.86	0.78	0.39	NA	NA	NA	NA
SVM (*RBF Kernel)*	0.90	0.96	0.88	0.83	0.84	0.29	0.86	0.87	0.86	0.83
Random Forest *(500 Trees)*	0.89	0.86	0.77	0.79	0.82	0.78	0.87	0.78	0.85	0.78
*Average*	0.87	0.89	0.79	0.81	0.77	0.51	0.84	0.82	0.82	0.80
B										

**TABLE 8:** ML algorithms prediction accuracy (RMSE) by the left (LH) and right (RH) brain hemispheres in five IGT blocks

	Block 1		Block 2		Block 3		Block 4		Block 5	
	LH	RH	LH	RH	LH	RH	LH	RH	LH	RH
Algorithm (10‐Folds CV)	SVM (RBF Kernal)	SVM (RBF Kernal)	ANN	ANN	SVM (RBF Kernal)	Random Forest (500 Trees)	Random Forest (500 Trees)	SVM (RBF Kernal)	SVM (RBF Kernal)	SVM (RBF Kernal)
RMSE	3.32	3.36	3.28	3.45	5.82	4.27	6.23	6.79	6.43	6.89

## 4 DISCUSSION

As noted in the previous research, the hemodynamic response to the stimulation of the decision‐making process in all five IGT blocks and both brain hemispheres has different patterns and differs significantly (Table 1). At the same time, thresholds between blocks are not clearly drawn. Eventually, the transfer of psychosomatic conditions of the experiment participants from total uncertainty and ambiguity in decision making at the beginning to certainty and risk acceptance at the end of each task session is not direct and smooth. All of these assumptions are reflected in the current study: findings of correlations and algorithm performance are substantially varied by IGT blocks and by using data from the left or the right brain hemispheres (Tables 5, 6, 7, and 8).

Generally, the current experiment findings do not contradict previous studies: the highest achieved correlation in average value is 0.24 (*t*(4) = 2.072, *p*‐value = 0.106) in the left hemisphere while performing the fourth 20 cards chooses. The correlation between HbO activation and IGT score was observed during experiments of Ono et al. (2015) and Li et al. (2019). But unlike them, dividing results not only by blocks as in the study of Li et al. (2019) and not just by hemispheres as in the study of Ono et al. (2015), current research allows weight and account for all correlations more precisely and reliable.

One more crucial specificity of the present work is the ability to distribute correlation by HbO signal features, not only mean value but standard deviation, variance, kurtosis, and skewness and result estimation by two methods: Pearson's and Spearman's. The participant's brain hemodynamic measured by fNIRS is not normally distributed signal during the whole task time; thus, signal shape parameters from the left hemisphere provide the highest achieved moderate positive correlation with the IGT score in block 4: 0.57 (Table 5b), that is outperforming previous findings in terms of blocks (0.45 in the study of Li et al., 2019) and in terms of brain hemispheres (−0.38 in the work of Ono et al., 2015). So, the “null” hypothesis should be rejected, and the question about spreading the correlation into the feature space could be marked as solved.

Consequently, ML models could exit beyond simple correlation, and evaluate and predict gaming behavior by participants’ brain activation during learning effect transfer from uncertainty to the risk. In total, 100 ML models were built separately in each IGT block and both PFC hemispheres (Tables 6a; Table 7a). The experiment determined that switching the holdout method from 70/30 to 80/20 and the CV method from fivefold to 10‐folds add more prediction accuracy by both performance metrics (Table 6b; Table 7b). SVM with RBF showed one of the best accuracies: lowest RMSE 3.37 – 7.84 (Table 6b) and highest R Squared 0.29 – 0.96 (Table 7b). Based on achieved RMSE and R Squared, the best fitted model was chosen in both hemispheres and each block (Table 8) and applied to the randomly selected testing dataset. As illustrated in Figure 5, predicted values marked in red are keeping learning effect based on classic IGT rules for healthy experiment participants—the task score increases from block 1 to block 5 by different curve patterns using HbO signal levels from the left and the right hemispheres as predicters.

## 5 CONCLUSION

Since its development from 1994 to the present, IGT remains a reliable tool for estimating human behavior during gaming. Adding to the experiment, fNIRS capabilities create a coherent flow of neuroimaging data that, combined with IGT and driven by ML, open new horizons and perspectives for scientists in investigating brain mystery and game‐related mental disorders.

In the current research, ML models were developed and applied to the testing dataset that allowed predicting IGT score behavior and general positive task trend. In terms of IGT rules, with the growth of experiment participants’ experience, the brain activation measured as HbO signal levels increases with the acceleration of the decision‐making process and gaming. Dividing the HbO signal by features allows identifying the strongest correlation between cognitive task performance and brain activation that was not investigated before. Participants add risk in decisions of card choices, and the standard deviation around mean value increases eventually. Inside the ML regression task, RMSE as a measure of prediction error increases accordingly. Thus, this reflects on a prediction of human behavior during gaming based on activity in the brain.

## CONFLICT OF INTEREST

The authors declare no conflict of interest.

### PEER REVIEW

The peer review history for this article is available at https://publons.com/publon/10.1002/brb3.2536
